# Lung volumetry of osteogenesis imperfecta type 3 subjects is not correlated with thoracic scoliosis and anthropometric data

**DOI:** 10.1186/s13023-025-03797-y

**Published:** 2025-06-02

**Authors:** Jorge Lopez-Beneyto, Elisabetta Ponte, Vicente Martínez-Sanjuan, Maria Fernandez-Velilla, Federico Mata-Escolano, Maribel Torres-Sanchez, Francisco García-Río, Shahed Nalla, Ilker Ercan, Juan A. Sanchis-Gimeno

**Affiliations:** 1 Facultat de Medicina i Odontologia, Universitat de València, Valencia, Spain; 2 Complejo Hospitalario de Toledo, Toledo, Spain; 3 ASCIRES Biomedical Group, Valencia, Spain; 4 La Paz University Hospital Biomedical Research Foundation, Madrid, Spain; 5 Faculty of Health Sciences, University of Johannesburg, Johannesburg, South Africa; 6 Bursa Uludağ Üniversitesi, Bursa, Türkiye

**Keywords:** Anthropometric data, Computed tomography, Lung volumetry, Osteogenesis imperfecta, Scoliosis

## Abstract

**Background:**

This study aimed to investigate the relationship between lung volumetry, thoracic scoliosis, and anthropometric data (height, weight, BMI) in patients with Osteogenesis Imperfecta (OI) Type 3. Three hypotheses were tested: H1 predicted lower lung volumes in patients with OI Type 3 compared to controls, H2 predicted differences between right and left lung volumes in patients with OI Type 3 due to chest deformities, and H3 predicted a correlation between lung volumes in patients with OI Type 3 and their thoracic scoliosis and anthropometric data.

**Methods:**

Age, biological sex, weight, height, body mass index (BMI), Cobb angle of thoracic scoliosis, left and right lung volumes, and total lung volume were recorded. CT scans were performed on all participants, and lung volumetry was analysed using specialised software. The intraclass correlation coefficient was used to assess measurement reliability, and statistical analysis was conducted to examine correlations between variables.

**Results:**

Patients with OI had significantly lower total lung volumes than controls (*p < *0.001). However, no significant correlation was found between lung volumetry and scoliosis (r =− 0.406; *p = *0.244), age (r = 0.201; *p = *0.578), height (r = 0.479; *p = *0.162), weight (r = 0.358; *p = *0.310), or BMI (r = − 0.042; *p = *0.907) in OI patients. In the control group, significant correlations were observed between lung volume and height (r = 0.756; *p = *0.011) and weight (r = 0.638; *p = *0.047).

**Conclusion:**

OI type 3 patients have lower lung volumes than healthy subjects, but have no left and right lung volume differences. In addition, they did not present any correlation between lung volumes and scoliosis, height, weight, and body mass index.

## Introduction

*Osteogenesis Imperfecta* (OI) is a group of hereditary disorders that impact the synthesis of type I collagen, the most abundant protein in the human body, especially the extracellular matrix of bone. The condition OI occurs in 1 in 15,000 to 20,000 births and affects 1 in 200,000 individuals, with no predominance of biological sex or race [1]. These disorders are primarily driven by autosomal dominant mutations in the genes encoding the alpha chains of type I collagen, COL1A1 and COL1A2 [2, 3].

OI presents a wide range of clinical severity, from fractures in utero to individuals with normal adult height and low fracture incidence. While recent advancements have introduced new types of OI syndromes [4], the conventional classification proposed consists of four main groups based on the variability of physical characteristics: Type I, Type II, Type III, and Type IV [5, 6].

Patients with type 3 OI develop short stature, multiple fractures, spinal deformities, cardiovascular insufficiency, and progressive bending of long bones [5–10], and is associated with a reduced life expectancy, with cardiopulmonary failure and respiratory infections being the primary causes of death; thus, prevention and treatment are crucial in prognosis [8, 10].

Several studies support that, structural deformities in the thoracic wall, such as spinal and rib cage deformities, may contribute to cardiopulmonary issues in severe OI [11–17]. These studies used the Cobb angle to measure the severity of scoliosis and correlate it with pulmonary function, observing that the greater the scoliosis, the greater the pulmonary involvement. Wekre et al. [18] and Norimatsu et al. [19] suggested that respiratory dysfunction might be secondary to progressive spinal deformity. Later, Keuning et al. defined that increased thoracic scoliosis correlated with decreased vital lung capacity in type 3 OI patients [20]. The group led by Lo Mauro et al. observed altered thoracic geometry in patients with type 3 OI, particularly in perimeter, area, and volume. They also assessed how severe structural deformities were compensated by abdominal expansion at rest and during maximum manoeuvre due to a restricted thoracic capacity [21]. Similarly, Sanchis-Gimeno et al. [22] conducted the first 3D geometric morphometric study of the thorax in osteogenesis imperfecta patients. They analyzed the skeletal thorax using 3D geometric morphometrics and found a significant relationship between rib shape and respiratory.

In most studies cited in the literature, simple radiography is the primary diagnostic technique used to study these patients. Despite its limitations, it is a cost-effective and highly reproducible technique. Some authors have employed computed tomography (CT) scans for a more detailed study of these patients [23].

CT has undergone significant evolution since its introduction in 1971. Initially used for obtaining axial brain images, it has become a versatile 3D technique for imaging various anatomical areas. Current CT technology provides high-quality images with improved spatial resolution and low contrast. Notable capabilities include obtaining 3D images and using applications to enhance diagnosis. CT remains a valuable tool in patient diagnosis and monitoring, but it should be evaluated and clinically justified in each case [24].

Previously, CT has been used in OI patients to study 3D assessment of craniofacial, dental, and upper respiratory anomalies in assessing microarchitecture, bone mass, and mineralization and evaluating response to antiresorptive treatments. Morphometric studies have also used it to correlate ribs with spirometry values [22, 25–27].

The other aspects researched are the potential relationship between pulmonary function and anthropometric measures (height, weight, and BMI), which has been investigated in non-OI patients. A significant correlation has been established between higher forced vital capacity (FVC) and forced expiratory volume in 1 s (FEV1), associated with greater height, weight, and body mass index (BMI) [28, 29].

In summary, to date, it is known that OI Type 3 patients present a relationship between the morphometry of the ribcage and spine and respiratory function. Still, there is insufficient information about the possible relationship between their anthropometric data (height, weight, BMI), thoracic scoliosis and lung volumetry. According to the evidence mentioned before, we formulate the following Hypotheses (H): H1 predicts that OI type 3 patients present lower lung volumes than control subjects; H2 predicts that OI type 3 patients present differences between the right lung and left lung volumes secondary to their ribcage deformities; and H3 that predicts that there is a correlation between the lung volumes of OI type 3 patients and their thoracic scoliosis and anthropometric data (height, weight, body mass index). Testing the formulated hypotheses may be a step forward in better understanding lung involvement in patients with OI.

## Methods

To test H1, H2, and H3, we recruited ten adult volunteers (six females, four males) classified with OI Type 3 by genetic testing. All of them were receiving bisphosphonate therapy. Ten non-smoking healthy adults (five females, five males) were selected as the control group. The study followed the tenets of the Declaration of Helsinki, was approved by the local Ethics Committee of the University of Valencia, Spain (approval n. H1417174744011), and each participant gave written informed consent for using their medical data.

Information such as age, sex, weight, height, BMI, Cobb angle of thoracic scoliosis, left lung volume, and right lung volume was obtained from OI and healthy subjects. Scoliosis was not measured in controls due to its absence among these subjects.

CT scans were performed on all participants in the supine position at full inspiration. CT scan studies were carried out in OI patients to analyse their clinical evolution, while in the control group, searching for possible neoplasms was undertaken, which were not found. Participation of all subjects was voluntary after being advised about the radiation exposure during CT scans.

### CT protocol and imaging evaluation

**Fig. 1:**
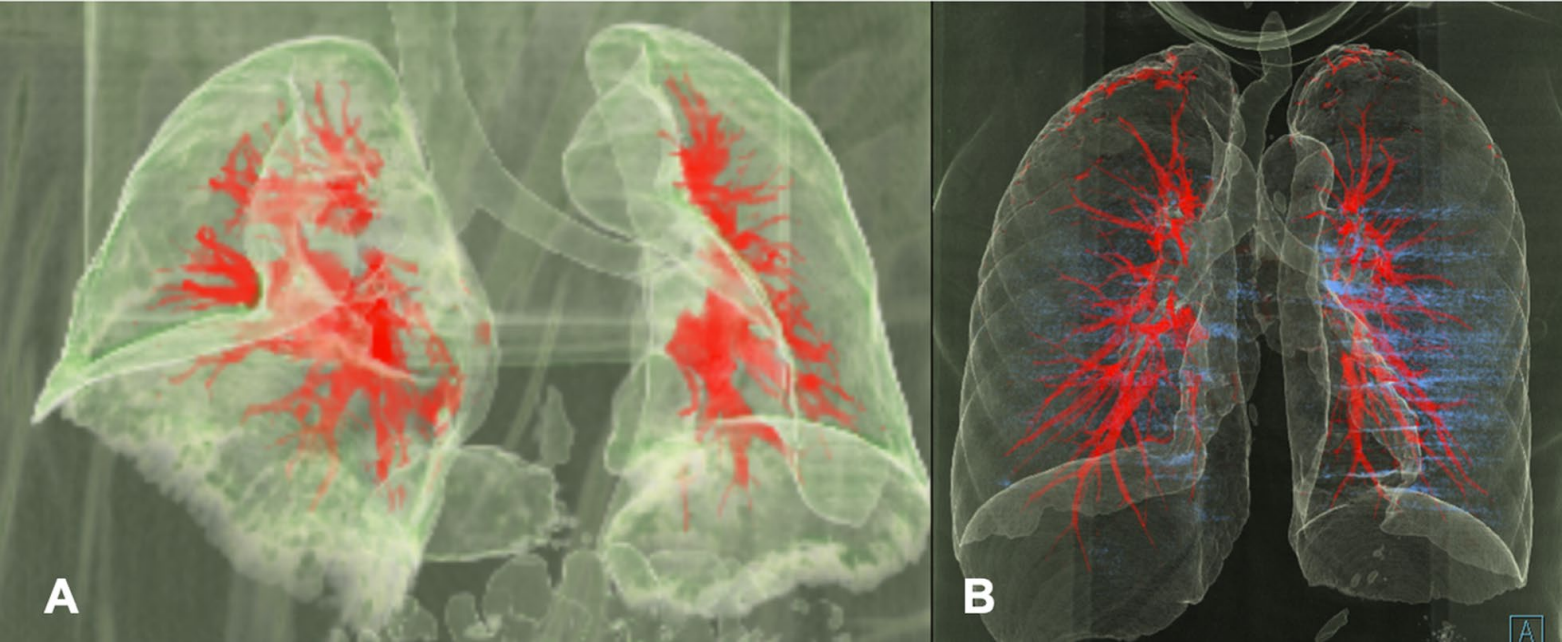
3D volumetric computed tomography reconstruction showing the lung parenchyma, trachea and pulmonary vascularization. **A**: Osteogenesis Imperfecta. **B**: Control group

**Fig. 2:**
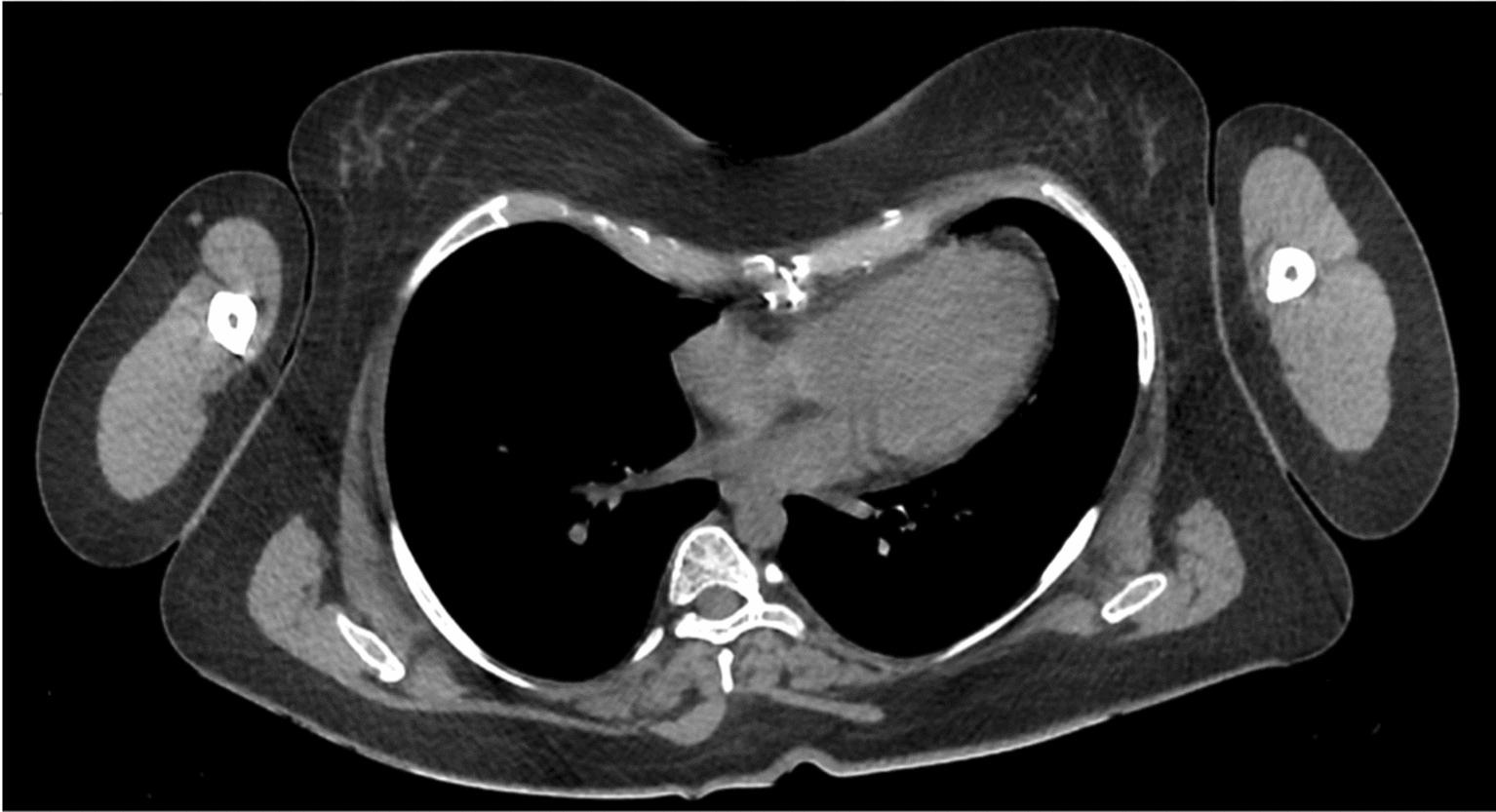
Axial computed tomography slice (inferior view) showing a rotated vertebral column protrusion into the right thoracic cavity in an Osteogenesis Imperfecta patient

A high-resolution volumetric chest CT scan was performed on all subjects in the study (Figs. 1 and 2). The protocol included a topogram to determine the boundaries of the volume to be acquired, ranging from the lung apices to the diaphragmatic domes, during maximum inspiration. The study was repeated during full expiration, both acquired in a cranio-caudal direction. No oral or intravenous contrast was administered. Patients were instructed in advance on performing respiratory maneuvers while lying on the CT table.

In OI patients, CT images were acquired using a GE MEDICAL SYSTEMS Discovery CT 750 HD scanner (Milwaukee, Wisconsin, USA). In our study, a helical scan with TOTAL BODY coverage was performed on the patient during inspiration. The radiological technique employed was 140 kV and 300 mAs. The images were acquired using the STANDARD filter and the LUNG and BONE algorithms. The slice thickness for each plane was 1.25 mm. Additionally, using the STANDARD and LUNG reconstruction algorithms, Multi-Planar Reconstruction images were obtained in both coronal and sagittal projections [30–32].

The control group used a 16-detector CT scanner (Somatom Emotion 16, Siemens Medical Solutions, Erlangen, Germany). Radiation parameters were adjusted conventionally based on each patient's morphological characteristics: 120 kV with a tube current of 160 mA. The used slice thickness was 0.75 mm, with a reconstruction increment of 0.5 mm, a pitch ratio in the spiral scan of 0.8, and a collimation thickness of 0.6 mm.

### Pulmonary volumetry

Lastly, we utilized the SYNGO VIA platform v. 8.7 (SIEMENS Healthineers, Erlangen, Germany) for semi-automatic volumetry with the 3D PULMO application [30–32]. The software Syngo.CT Pulmo 3D was used to analyze volumetric CT datasets focusing on pulmonary assessment. This application can examine the lungs as a whole or in specific sections, detecting areas with Hounsfield Unit values above or below a predefined threshold. Statistical techniques such as histogram analysis and percentiles are applied to conduct this assessment. With Syngo.CT Pulmo 3D, it is possible to perform a detailed analysis of lung tissue and airways. Both the OI and control groups were analysed using the same program.

For measurement of the scoliotic curvature, we produced reconstructed digital 2D images of the thoracic spines in the mid-sagittal plane from each of the subject’s CT scans; we have done this with Multi-Planar Reconstruction and 3D Volume Rendering images in an attempt to be as accurate as possible and applied the same technique as from a conventional radiograph to measure the Cobb angle [33] chosen the inferior endplate of the caudal vertebra of the thoracic curve and the superior endplate of the cranial vertebra as inferior and superior limits. The Cobb angle measurement was omitted for patients within the control group, as scoliotic conditions were not evident.

To evaluate the reliability and validity of the thoracic scoliosis and the lung volume measurements, the same researcher measured the Cobb angle and the lung volumes three times, and the intraclass correlation coefficient (ICC) was estimated. ICC values less than 0.5 are considered to be indicative of poor reliability, values between 0.5 and 0.75 indicate moderate reliability, values between 0.75 and 0.9 indicate good reliability and values greater than 0.90 indicate excellent reliability [34].

Non-parametric tests were performed for the statistical analysis. Fisher’s exact and Mann–Whitney U tests were used to compare continuous variables between group comparisons for categorical variables. Descriptive statistics are provided as median (min–max) for continuous variables and frequency and percentage for categorical variables. The correlation between the variables was examined with the Spearman rank correlation coefficient. α = 0.05 was considered statistically significant. Data were analysed using the IBM SPSS Statistics (Version 25).

## Results

Tables 1 and 2 present the values obtained in the subjects analysed. Table 2 revealed significantly lower lung volumes in OI patients but no significant differences between the left and right lung volumes in both OI (*p = *0.589) and control subjects (*p = *0.187).

**Table 1:** Comparison between Osteogenesis Imperfecta and Healthy (control) subjects

	Osteogenesis Imperfecta	Control subjects	
	Median (min–max)	Median (min–max)	*p-*value
Age (years)	39.5 (19.0–57.0)	53.0 (46.0–69.0)	0.009 *
Height (cm)	119.0 (100.0–145.0)	167.0 (153.0–182.0)	< 0.001 *
Weight (Kg)	46.5 (30.0–70.0)	71.5 (50.0–106.0)	0.001 *
BMI (kg/m^2^)	32.5 (19.9–55.0)	24.6 (21.4–33.8)	0.011 *
Cobb scoliosis angle (º)	29.8 (19.9–42.3)	–	–
FVC (L)	2.4 (1.0–4.0)	3.7 (2.7–5.8)	0.005 *
FEV1 (L)	2.0 (1.0–3.4)	3.0 (2.0–4.4)	0.015 *
FVC, % pred (%)	72.9 (53.5–93.4)	112.1 (90.8–126.0)	< 0.001 *
FEV1, % pred (%)	75.9 (60.2–108.4)	109.8 (85.5–129.9)	< 0.001 *

FVC: forced vital capacity; FEV1: forced expiratory volume in 1 s, FVC% pred: forced vital capacity expressed as percentage of predicted value; FEV1% pred: forced expiratory volume in 1 s expressed as percentage of predicted value. * Statistically significant

**Table 2:** Lung volumes in Osteogenesis Imperfecta and Healthy control subjects (ml)

	Osteogenesis Imperfecta	Control subjects	
	Median (min–max)	Median (min–max)	*p-*value
Left lung volume	827.5 (341–1552)	2187 (1689–3491)	< 0.001 *
Right lung volume	1112.5 (410.0–1812)	2549 (1917–3690)	< 0.001 *
*p-*value	0.569	0.187	

^*^Statistically significant

The ICC used to assess measurement accuracy for the thoracic scoliosis measurements was 0.982, with a CI95% from 0.961 to 0.996, which reflects excellent reliability. In contrast, the ICC used to assess measurement accuracy for lung volume measurements was 0.992, with a CI95% from 0.987 to 0.997, reflecting excellent reliability.

We found no significant correlation between the left lung volume and the age (r = 0.255; *p = *0.476), height (r = 0.215; *p = *0.128), weight (r = 0.491; *p = *0.150), BMI (r = − 0.090; *p = *0.803), and scoliosis (r = − 0.515; *p = *0.128) in OI patients. The same was observed between the right lung volume and age (r = 0.109; *p = *0.763), height (r = 0.515; *p = *0.127), weight (r = 0.321; *p = *0.365), BMI (r = − 0.030; *p = *0.934) and scoliosis (r = − 0.430, *p = *0.215).

In the control group, no significant correlation was found between left lung volume and age (r = − 0.197; *p = *0.586) and BMI (r = 0.261; *p = *0.467), while it was significant between left lung volume and height (r = 0.762; *p = *0.010) and weight (r = 0.711; *p = *0.021). Again, the correlation was no significant in the control group for the right lung volume and age (r = − 0.234, *p = *0.516) and BMI (r = 0.267, *p = *0.466), but it was significant for weight (r = 0.681; *p = *0.032) and height (r = 0.774; *p = *0.009).

Regarding the total lung volume of the subjects (left lung plus right lung volumes), OI patients had significantly lower values than controls (*p < *0.001). In addition, when considering the total lung volume of the subjects, we found no significant correlation with age (r = 0.201; *p = *0.578), height (r = 0.479; *p = *0.162), weight (r = 0.358; *p = *0.310), BMI (r = − 0.042; *p = *0.907), and scoliosis (r = − 0.406; *p = *0.244) in OI patients. Nevertheless, in the control group, no significant correlation was found between the total lung volume and age (r = − 0.258; *p = *0.471) and BMI (r = 0.176; *p = *0.627). We did, however, find a significant positive correlation between the total lung volume and height (r = 0.756; *p = *0.011) and weight (r = 0.638; *p = *0.047).

## Discussion

The relationship between the Cobb angle in scoliosis and lung function has been the subject of extensive medical literature research involving multiple disciplines. Scoliosis, characterized by an abnormal 3D curvature of the spine, raises questions about how this morphological alteration can affect respiratory mechanics and, ultimately, lung function [35, 36]. The Cobb angle, an essential clinical measure, is used to quantify the magnitude of the spinal curvature in scoliosis patients. Research has revealed that as the Cobb angle increases, especially in curves affecting the thoracic region, significant biomechanical stresses are generated that affect the respiratory system [37]. As it is known, the spine's altered anatomy in scoliosis can directly affect lung function as vertebral deformities can change the shape of the ribcage and rib positions. These anatomical changes can reduce the lungs' ability to expand during inhalation, decreasing vital capacity and total lung volume [38]. It is also important to note that the effects of the Cobb angle on lung function can vary depending on the location of the curvature in the spine. Due to their proximity to the ribs and ribcage, thoracic curves may have a more pronounced impact on respiratory mechanics than lumbar curves. Additionally, curves affecting the upper thoracic spine region may significantly affect lung function more than lower curves [39]. Regarding scoliosis and lung volumes, Adam et al. [40] showed that both left and right lung volumes decreased in scoliosis patients compared to healthy subjects. However, the decrease was more pronounced in the left lung volume, resulting in a greater difference in lung volumes between the left and right sides in scoliosis patients [40]. Nevertheless, we have found no significant differences between the left and right lung volumes in our OI patients, although all presented scoliosis.

In the context of OI, this takes on particular significance as OI patients are more likely to develop scoliosis due to bone fragility. The Cobb curve refers to measuring the spinal column deformity angle in scoliosis. As this curve becomes more pronounced, it can exert pressure on the thoracic cavity, further reducing lung capacity and impeding respiratory function [11–17]. This biomechanical impact on the respiratory system can lead to breathing difficulties, decreased oxygenation of the blood, and increased susceptibility to respiratory infections in OI patients with severe scoliosis. Kaplan et al. [41] achieved highly effective results in patients with thoracic insufficiency syndrome due to OI type III, using a spino-thoracic fixation device that improved the Cobb angle and lung function [41]. Therefore, understanding this relationship between the severity of scoliosis (measured by the Cobb angle) and biomechanical stresses on the respiratory system is essential for the comprehensive care and management of individuals with OI, as it can influence clinical decisions and treatment strategies for addressing scoliosis and pulmonary abnormalities. Previously, Gollogly et al. [42] determined right and left lung volume values and total lung volume by 3D reconstruction of CT in a similar manner to that we have made, while Wen et al. [43] observed decreased lung volumes in scoliosis patients. In this context, and although our OI type 3 patients presented scoliosis, we have not found any association between lung volumetry and the severity of scoliosis of OI patients.

On the other hand, obesity is associated with several alterations in lung function. It is related to a reduction in lung volumes. This reduction is primarily due to the mechanical effects of excess body fat on the chest wall and diaphragm, resulting in a decrease in vital and functional residual capacity. Moreover, obese individuals often need to exert extra effort to breathe due to the increased resistance offered by excess body fat, leading to reduced exercise tolerance. Obesity can also alter the mechanical properties of the respiratory system, affecting lung compliance and resistance, which in turn influences lung function [44]. Additionally, height plays an important role in determining lung volume. Generally, taller individuals have larger lung volumes compared to shorter individuals. This relationship is due to the thoracic cavity's size, which is influenced by skeletal dimensions, including height. Furthermore, taller individuals usually have larger tidal volumes, which are important for adequate ventilation [29]. Due to their short stature and inability to engage in physical activity, OI patients often experience overweight or even obesity in some cases, which can cause alterations in lung function [45]. Therefore, we evaluated how height, weight, and BMI individually could affect total lung volume in OI patients. However, no significant correlations were found in OI patients.

Another element to consider is the impact of pulmonary manifestations on the quality of life of OI patients. The systematic review by McDonald et al. [46] observed that intrinsic and extrinsic lung disease is common in OI and represents a significant source of morbidity and mortality (i.e. dyspnoea, impaired sleep, fatigue and frequent respiratory tract infections). The authors also noted that OI patients present limited aerobic capacity and exercise tolerance, contributing to difficulties with daily activities. In sum, the presence of cardio-pulmonary comorbidities was associated with lower health-related quality of life in OI patients.

One limitation of this study was the small number of included patients. OI is a rare disease, and additionally, we analyzed a subtype of OI, thus making it inherently difficult to recruit many participants, as happens with research studies carried out before by different authors [14, 16, 17, 21, 22, 47–53]. The study on OI patients with significant deformities and, in some cases, metallic fixation material and the need for some patients to use wheelchairs faced limitations that should be considered when interpreting its results. However, the study provides valuable information and a more comprehensive view of the disease. Additionally, including male and female patients in the study makes it more representative of the population affected by this disease, increasing its applicability.

Regarding the assessment of lung parenchyma using CT, additional challenges arose. Some patients had difficulty entering the CT machine due to their deformities or the metallic fixation material in their vertebral bodies. This could have led to a limitation in the quality of the obtained images and, therefore, a less accurate evaluation of pulmonary health, since metallic fixation material can generate artefacts in CT images, further complicating the study evaluation and the identification of potential pathologies. Some patients could not be properly positioned supine during the procedure, which may have further affected the image quality and the ability to assess pulmonary pathology accurately. Additionally, the assessment of lung volumetry was affected by deformities in some cases. The program used for this purpose had to be reviewed and adapted to accommodate the specific deformities of the patients, which could have introduced some degree of variability in the results. Another limitation was the difficulty in measuring the Cobb angle, a parameter commonly used to evaluate spinal deformities. Since our patients had significant spinal deformities, the precise measurement of this angle may have been challenging or inaccurate, impacting the study's ability to assess the severity of spinal deformities. However, we could adequately measure the Cobb angle with the reconstructed 2D digital images of the thoracic spines in the mid-sagittal plane from each of the subjects' CT scans. Nevertheless, despite limitations in the number of patients and technical difficulties associated with evaluating pulmonary and spinal pathology in this OI population, we believe this study provides valuable information that contributes to understanding the disease and underscores the need to address these challenges in future research.

## Conclusions

Our research found that OI type 3 patients have lower lung volumes than healthy subjects (H1 confirmed). Also, we have detected that there are no left or right lung volume differences in OI type 3 patients (H2 discarded) and that in these patients, there is no correlation between lung volumes and scoliosis, height, weight, and body mass index (H3 discarded).

## Data Availability

The datasets generated and/or analyzed during this study are not publicly available, as CT data and DICOM headers contain patient information. Data can be obtained on reasonable request from the corresponding author.

## Abbreviations

OI: Osteogenesis imperfecta
CT: Computed tomography
BMI: Body mass index
ICC: Intraclass correlation coefficient
